# Stability-Indicating UPLC Method for Tramadol HCl Impurities in the Tramadol Injection after Dilution by Infusion Fluids (5% Dextrose and 0.9% Sodium Chloride)

**DOI:** 10.3797/scipharm.1305-20

**Published:** 2013-07-14

**Authors:** Anil K. Binnor, Khagga Mukkanti, Mulukutla V. Suryanarayana, Sunilendu B. Roy

**Affiliations:** 1 Pharmaceutical Technology Center, Cadila Healthcare Ltd, Moraiya, Ahmedabad-382210, Gujarat, India.; 2 Center for Chemical Sciences and Technology, IST, J.N.T. University, Kukatpally, Hyderabad-500072, A. P., India.; 3 J. N. T. University affiliated research guide, Kukatpally, Hyderabad-500072, A. P., India.

**Keywords:** Stability-indicating UPLC method, Tramadol HCl impurities, 5% Dextrose injection, 0.9% Sodium Chloride injection, Validation, Stability study

## Abstract

A novel, rapid, and sensitive ultra-performance liquid chromatography (UPLC) method has been developed and validated as per ICH guidelines for the determination of tramadol HCl impurities in the tramadol HCl injection after reconstitution by infusion fluids (5% dextrose and 0.9% sodium chloride). The tramadol HCl injection is for the treatment of patients with moderate-to-severe pain. The stability of the reconstituted solution is critical before intravenous injection. The literature search resulted in few published articles on assays of tramadol in infusion fluids by conventional HPLC. No attempts have yet been made to determine the impurities in infusion fluids, as the concentration of tramadol after reconstitution is extremely low (0.4 mg/mL) and that of impurities is even lower. The proposed method is novel as it allows the quantitation of the impurities of tramadol HCl and is based on modern chromatographic techniques like UPLC. The method was developed using the Waters Acquity BEH C18 column with a mobile phase consisting of a gradient mixture of solvent A (trifluroacetic acid buffer) and solvent B (methanol: acetonitrile). The model stability study was designed by diluting the tramadol HCl injection in the 5% dextrose injection and 0.9% sodium chloride injection. Each mixture was kept under storage at room temperature (25 ± 2°C) for testing at initial, 2, 4, 8, 12, 18 & 24 hours. The validation study illustrates that the proposed method is suitable for the determination of tramadol and its impurities. The proposed method makes use of the LC-MS-compatible mobile phase. It can be useful for the determination of tramadol HCl and its impurities in plasma samples and other pharmaceutical dosage forms.

## Introduction

**Fig. 1:**
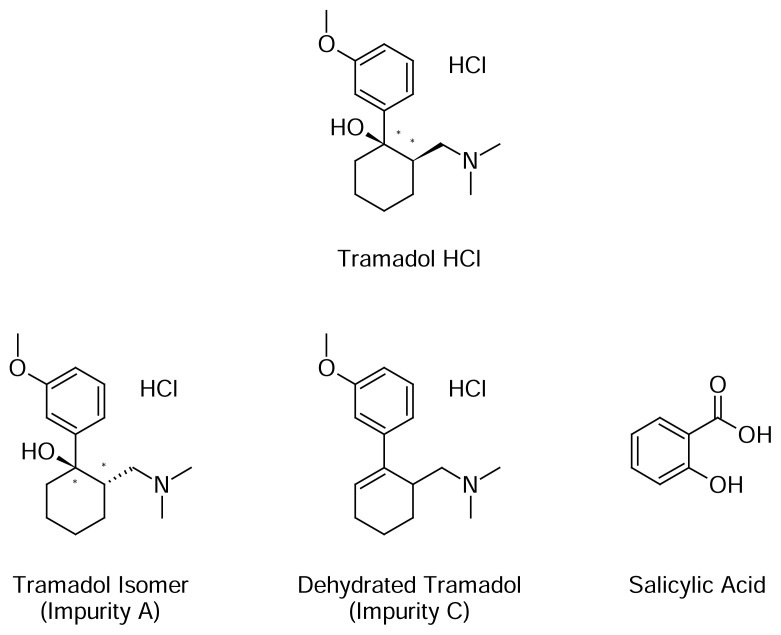
Structures of tramadol hydrochloride and its impurities

Tramadol hydrochloride is a centrally-acting synthetic opioid analgesic that binds to specific opioid receptors. The chemical name for tramadol hydrochloride is *rac*-(1*R*,2*R*)-2-[(dimethylamino)methyl]-1-(3-methoxyphenyl)cyclohexanol hydrochloride (Fig 1). Its structural formula is C_16_H_25_NO_2_·HCl. The molecular weight of tramadol hydrochloride is 299.8. Tramadol hydrochloride is a white crystalline powder that is freely soluble in water and ethanol.

In the published literature, limited LC methods have been reported for the determination of tramadol HCl. The assay methods reported pertain to its compatibility with few selected drugs [1], long-term stability in 5% dextrose [2–4], and stability with haloperidol for continuous subcutaneous infusion [5]. The assay methods are also reported in an ample dosage form [6], in oral drops [7], and by TLC and densitometry [8]. The simultaneous estimation with paracetamol and domperidone in tablet dosage form is reported [9]. Simultaneous estimation methods of tramadol along with metabolites in human plasma are reported [10–15]. The assay methods for tablets are reported by Kamble [16] and Zaghloul [17]. The assay method for capsules is reported by Kartinasari [18]. We have developed a method to determine impurities in infusion fluids. It was a challenging task as the concentration of tramadol HCl after dilution is extremely low (0.4 mg/mL) and that of impurities is even lower. The proposed method is novel as it utilizes modern techniques like UPLC and allows the quantitation of impurities in the tramadol HCl injection in infusion fluids. The method was developed using the Waters Acquity BEH C18 column with a mobile phase consisting of a gradient mixture of solvent A (trifluroacetic acid buffer) and solvent B (methanol: acetonitrile).

The tramadol HCl injection is currently marketed in vials at a dosage strength of 100 mg (2 ml ampoule). A series of stability studies was conducted to evaluate the stability of the tramadol HCl injection after its dilution. The method was validated as per ICH guideline Q2 (R1) [19] over the concentration range after dilution (i.e. concentration of 0.4 mg/ml).

The proposed method makes use of a photodiode array detector (DAD) as a tool for peak identity and purity confirmation. The peaks of tramadol and its impurities are homogenous and there are no co-eluting peaks of the impurities observed by peak purity analysis. The method development, method validation, and model stability testing experiments are described in this article.

## Experimental

### Chemicals and Reagents

Tramadol HCl (lot WS107K0, purity 99.5% w/w), tramadol impurity A (lot 08/TM-IMPURITY-A/654/033, purity 99.09% w/w), impurity C (lot 08/TM-IMPURITY-C/654/034, purity 98.79% w/w), salicylic acid (lot B05G421PUR, purity 99.3% w/w), and tramadol HCl injection (lot MK 3452) were obtained from Cadila Healthcare Ltd (Ahmedabad, Gujarat). All chemicals required to perform the analytical research were obtained in analytical grade and solvents were HPLC grade reagents from Merck. High-purity water was obtained by using the Millipore Milli-Q (Milford, USA) purification system.

### Instruments

The pH of the solutions was measured by using a LABINDIA pH meter (PICO+). The Acuity UPLC™ system (Waters, Milford, USA) that was used consisted of a sample manager, a binary solvent manager, and photodiode array (PDA) detector. The chromatographic data were acquired and processed using Empower software. The intermediate precision (ruggedness) was performed on another Acquity UPLC™ system (Waters, Milford, USA) consisting of a sample manager, binary solvent manager, and tunable ultraviolet detector.

### Preparation of Stock Solutions

The stock solution of tramadol HCl at 0.4mg/ml was prepared by dissolving an appropriate amount in the dextrose and sodium chloride solutions. Working solutions were prepared from the above stock solution for the impurity determinations. A stock solution of the impurities (impurity A, impurity C, and salicylic acid) at 0.2 mg/ml were also prepared in the respective diluents. Validation study solutions for precision, linearity, accuracy, specificity, the limit of detection, and the limit of quantification were prepared using these stock solutions.

### Preparation of Reconstituted Solutions in the 5% Dextrose Injection and 0.9% Sodium Chloride and Storage

Two vials of the tramadol HCl injection at 100 mg-strength (2ml ampoule) were separately taken and reconstituted with 10ml each of 5% dextrose and 0.9% sodium chloride. This reconstituted solution was then transferred to two separate 250ml volumetric flasks and diluted to volume with respective diluents. The concentration of tramadol HCl in each solution was approximately 0.4 mg/ml. A quantization of tramadol HCl and its impurities was made immediately after the preparation of each solution and subsequent assays were performed after storage for 4, 8, 12, 18 & 24 hours at room temperature 25°±2°C.

## Results and Discussion

### Method Development of UPLC Method

The reversed-phase ultra-performance liquid chromatography (UPLC) method was developed by taking into consideration the aqueous solubility of tramadol HCl and UV absorbance maxima at 270nm. The mobile phase was a gradient mixture of solvents A and B. A 0.2% trifluoroacetic acid buffer was used as solvent A and a mixture of methanol and acetonitrile in the ratio 75:25, v/v; was used as solvent B. The mobile phase was filtered through a nylon 0.45 μm membrane filter. The gradient elution mode was used with varying concentrations of the organic mixture to modify the polarity of the mobile phase. The gradient program (T/%B) was set at 0/20, 15/60, 16/20, and 20/20. The reversed-phase chromatography uses a non-polar stationary phase. The octadecylsilane (C18) column of the brand Acquity BEH C18 with 100mm length and 2.1mm diameter was used. The particle size of the stationary phase was 1.7μ. The column temperature was kept at 30°C. The flow rate was kept at 0.20 ml/min. The injection volume of 10 μl was used with the full loop option.

The known impurities of tramadol HCl like the tramadol isomer, *rac*-(1*R*,2*R*)-2-[(dimethylamino)methyl]-1-(3-methoxyphenyl)cyclohexanol hydrochloride (impurity A), dehydrated tramadol, *rac*-1-[(1*R*)-2-(3-methoxyphenyl)cyclohex-2-en-1-yl]-*N*,*N*-dimethyl-methanamine hydrochloride (impurity C), and *o*-hydroxybenzoic acid (salicylic acid) were studied for their precise, accurate, linear, and specific determinations along with other unknown degradation impurities detectable by the proposed method. Fig. 1 shows the structures of tramadol HCl and its three impurities.

### Method Validation

The proposed UPLC method was validated as per ICH guideline Q2 (R1) [19] for performance characteristics like accuracy, precision, ruggedness, specificity, robustness, linearity, limit of detection (LOD), and the limit of quantitation (LOQ) as it determines the tramadol HCl impurities in a dilute solution.

### Precision

**Fig. 2:**
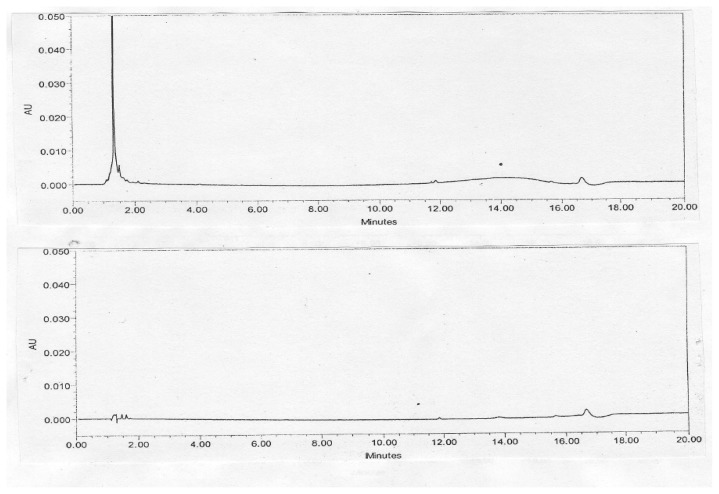
Typical Chromatogram of 5% Dextrose and 0.9% Sodium chloride

**Fig. 3:**
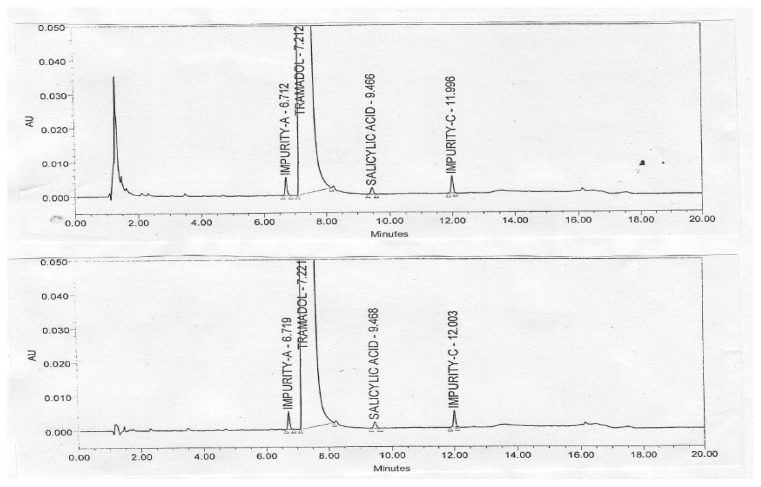
Typical Chromatogram of Tramadol Hydrochloride Sample Spiked with Impurities, in 5% Dextrose and 0.9% Sodium chloride

The precision of the method was verified by repeatability in a single day and the intermediate precision by different analysts on different days using different instruments. Repeatability and intermediate precision were performed by analyzing six individual preparations of the tramadol HCl injection spiked with 0.2% of its three impurities. The % RSD for the content of tramadol and its impurities was calculated. The results are summarized in the table. Fig 2 illustrates the typical chromatogram of the 5% dextrose and 0.9% sodium chloride injections. Fig 3 illustrates the typical chromatogram of impurities spiked in the tramadol HCl injection with 5% dextrose and 0.9% sodium chloride injections. The precision study results are summarized in Tables 1 & 2.

### Linearity

The method was evaluated for its ability to produce a linear correlation between the concentration of the analyte to be constituted and area response. Linearity test solutions for the method were prepared by diluting stock solutions of tramadol HCl and its impurities to the required concentrations. The solutions were prepared at six concentration levels from the LOQ to 120% of the specification level (LOQ=0.05, 0.075, 0.10, 0.20, and 0.25 %). The linearity calibration plots were obtained over the concentration range studied and the coefficient of correlation was greater than 0.999, which shows that excellent correlation existed between the peak area and concentrations of tramadol HCl and its impurities. The regression analysis results are summarized in Tables 1 & 2.

### Limit of Detection (LOD) and Limit of Quantification (LOQ)

**Fig. 4:**
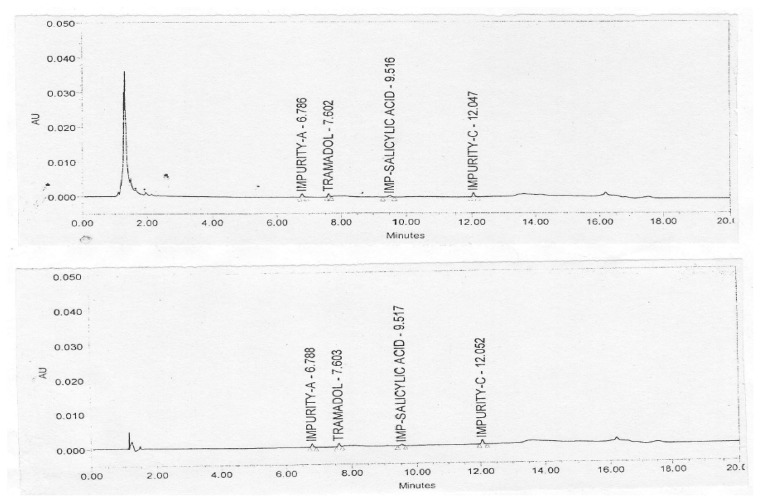
Typical Chromatogram of Tramadol Hydrochloride and its Impurities at LOQ Level in 5% Dextrose and 0.9% Sodium Chloride

The limit of detection of the method is the lowest detectable concentration of the method. The limit of quantification of the method is the lowest concentration of the method which can be quantitated with accuracy, precision, and linearity. The LOD and LOQ concentrations were determined by injecting a series of dilute solutions of tramadol HCl and its impurities. As per ICH guidelines, the reporting threshold for impurities is 0.05%. Hence, the limit of quantification was considered as 0.05% and limit of detection was considered as 0.025% (lower than the LOQ) based on the area response observed for the impurity peaks (response and visual inspection method). The precision and accuracy study was carried out at the LOQ concentrations of tramadol HCl and its impurities. Fig 4 illustrates the typical chromatogram obtained for the LOQ solutions in 5% dextrose and 0.9% sodium chloride. The results of the LOD and LOQ study are summarized in Tables 1 & 2.

### Specificity

The specificity of a method is the ability of a method to quantitate the analyte in the presence of its potential impurities. The specificity of the proposed method was carried out by performing stress studies and peak purity analysis by a photodiode array (PDA). Stress studies were carried out with UV light (254 nm), heat (100°C), acid (0.5 N HCl at 50°C), alkali (0.5 N HCl at 50°C), oxidative (3% H_2_O_2_ at 40°C), and moisture hydrolysis (50°C) to evaluate the ability of the proposed method to separate the tramadol peak from its degradation products. All stress study samples were analyzed at a 0.4mg/ml concentration of tramadol HCl. The stress study concludes that tramadol HCl is stable under all stress conditions i.e. acid and alkali hydrolysis, moisture hydrolysis, oxidative, heat, and photolytic (UV) conditions. The peak purity analysis by the photodiode array detector shows a purity angle below the purity thresholds and purity flag pass. Only one unknown impurity at a relative retention time of 0.93 was observed at the 0.02% level and the other impurities were observed below 0.02%. The blank diluent solutions and placebo solutions showed no interference at the retention times of tramadol and its impurities.

### Accuracy

The accuracy of the method was studied by conducting the standard addition and recovery experiments on infusion samples to determine tramadol impurities. The study was carried out in triplicate using four concentration levels: LOQ, 50%, 100%, and 150% of impurities with respect to the specification levels (i.e. 0.2% with respect to 0.4mg/ml). The % recovery values for the impurities are reported in Tables 3 & 4.

### Robustness

The robustness of a method is the ability of a method to remain unaffected by deliberate changes in method parameters. The critical parameters of flow rate and temperature were changed to assess the method performance. The robustness data analysis (retention times (RT) of tramadol and impurity peaks) shows that the method is robust to changes and suitable for its intended purpose. The results of the robustness study are reported in Table 5.

### Measurement of pH

The pH of the solution was determined using a glass combination electrode, which was calibrated. The pH measurement was performed immediately after the preparation of the solutions and on each storage point sample for up to 24 hours. The pH results are summarized in Table 6.

### Analysis of Tramadol HCl injection in 5% Dextrose and 0.9% Sodium Chloride

Tramadol HCl impurities were quantitated using a gradient reversed-phase ultra-performance liquid chromatography (UPLC) method. The diluted tramadol HCl standard injection (0.2% concentration with respect to the test solution concentration) was injected six times to check the system suitability performance. In this test, the tramadol HCl peak area response for the six injections was precise within 5% of the relative standard deviation. The standard injections were followed by tramadol HCl injections in the 5% dextrose injection and 0.9% sodium chloride injection for the respective storage stability time points of initial, 2, 4, 8, 12, 18 & 24 hours.

The concentration of the tramadol HCl impurities (expressed as a %) in each sample was calculated against the diluted working tramadol HCl standard area response. The relative response factors (RRF) of 0.95 for impurity A, 0.74 for impurity C, and 1.45 for salicyclic acid were used for the calculations to correct the area responses.

**Fig. 5:**
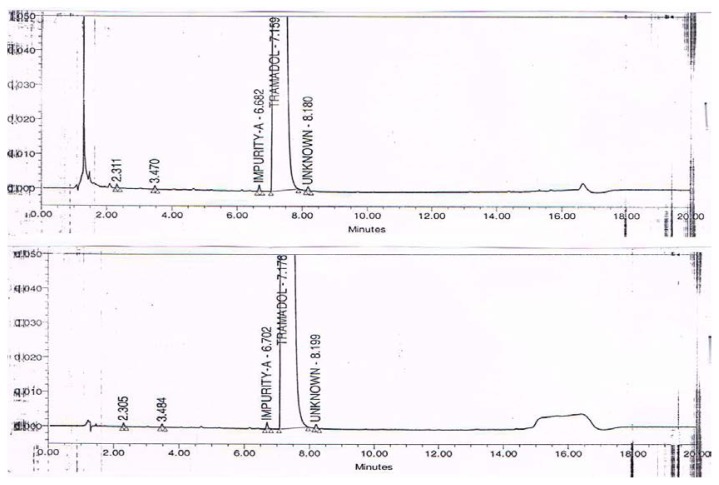
Typical Chromatogram of Tramadol Hydrochloride Injection in 5% Dextrose and 0.9% Sodium chloride

The results of the stability studies in each 5% dextrose injection and 0.9% sodium chloride injection solutions are summarized in Tables 7 and 8. Fig. 5 illustrates the typical chromatogram obtained for the tramadol hydrochloride injection in 5% dextrose and 0.9% sodium chloride. The concentration of tramadol HCl in all media was well above 98% by the end of the 24-hour storage period.

## Conclusion

The sensitive and rapid RP-UPLC method developed for the quantitative analysis of tramadol HCl impurities in the tramadol HCl injection after dilution by the infusion fluids (5% dextrose and 0.9% sodium chloride) is precise, linear, accurate, specific, and robust. The method is sensitive in terms of the low limit of detection and the limit of quantitation values as per ICH guideline Q2 (R1). The method is stability-indicating as indicated by the forced degradation studies and can be used for evaluating the stability of the tramadol HCl injection after dilution by infusion fluids (5% dextrose and 0.9% sodium chloride). The proposed method makes use of the LC-MS-compatible mobile phase. It can be useful for the determination of tramadol HCl and its impurities in plasma samples and other pharmaceutical dosage forms.

## Figures and Tables

**Tab. 1:** LOD, LOQ, Precision, and Regression Analysis in 5% Dextrose

Parameter	Tramadol HCl	Impurity A	Impurity C	Salicylic Acid
LOD (μg/mL & %)	0.099	0.099	0.099	0.099
	0.0247%	0.0247%	0.0247%	0.0247%
LOQ (μg/mL & %)	0.199	0.198	0.198	0.199
	0.0497%	0.0495%	0.0495%	0.0497%

Regression analysis				

Slope(b)	0.0002	0.0002	0.0003	0.0001
Intercept(a)	266.39	66.948	13.494	241.3
Correlation Coefficient	0.999	0.999	0.999	0.999
% Bias at 100% level	1.67	0.039	0.057	2.42

Precision analysis				

Precision (RSD %), N=6	0.0052	0.4394	0.6436	1.2904
Intermediate Precision (RSD%), Ruggedness, N=6	0.0138	0.7979	3.5008	1.4629

**Tab. 2:** LOD, LOQ, Precision, and Regression Analysis in 0.9% Sodium Chloride

Parameter	Tramadol HCl	Impurity A	Impurity C	Salicylic Acid
LOD(μg/mL)	0.099	0.099	0.099	0.099
	0.0247%	0.0247%	0.0247%	0.0247%

LOQ(μg/mL)	0.199	0.198	0.198	0.199
	0.0497%	0.0495%	0.0495%	0.0497%

Regression analysis				

Slope(b)	0.0002	0.0002	0.0003	0.0001
Intercept(a)	317.22	92.862	20.948	181.94
Correlation Coefficient	0.999	0.999	0.999	0.999
% Bias at 100% level	1.98	0.057	0.090	1.76

Precision analysis				

Precision (RSD %), N=6	0.0041	0.2170	0.4925	1.5485
Intermediate Precision (RSD%), Ruggedness, N=6	0.0041	1.5821	0.8571	6.8936

**Tab. 3:** Evaluation of Accuracy in 5% Dextrose

Spiked (%)	% Recovery in 5% Dextrose		
	
	Impurity A	Impurity C	Salicylic Acid
LOQ	105.02 ± 2.87	107.95 ± 0.98	101.23 ± 2.47
50%	114.71 ± 2.63	102.75 ± 0.31	103.78 ± 2.41
100%	109.71 ± 0.41	107.54 ± 0.08	101.48 ± 0.47
150%	110.95 ± 1.06	107.28 ± 0.30	103.47 ± 1.26

**Tab. 4:** Evaluation of Accuracy in 0.9% Sodium Chloride

Spiked (%)	% Recovery in 0.9% Sodium Chloride		
	
	Impurity A	Impurity C	Salicylic Acid
LOQ	104.04 ± 1.50	110.91 ± 1.08	109.45 ± 7.79
50%	106.79 ± 1.92	104.28 ± 0.34	106.03 ± 0.92
100%	104.54 ± 0.63	108.79 ± 1.69	99.38 ± 0.46
150%	108.21 ± 0.58	107.98 ± 0.37	105.53 ± 1.26

**Tab. 5:** The Results of Robustness Study

Robustness	Impurity A (RT, mins)	Tramadol (RT, mins)	Salicyclic Acid (RT, mins)	Impurity C (RT, mins)
Flow 0.18mL/min	6.717	7.222	9.463	12.002
Flow 0.20mL/min	6.697	7.198	9.452	11.982
Flow 0.22mL/min	6.669	7.148	9.365	11.923
Temperature 25°C	6.716	7.220	9.464	12.002
Temperature 30°C	6.697	7.198	9.452	11.982
Temperature 35°C	6.706	7.192	9.448	11.987

**Tab. 6:** pH Measurement in 5% Dextrose and 0.9% Sodium Chloride

Time (hours)	pH in 5% Dextrose	pH in 0.9% Sodium Chloride
0	5.95	6.11
2	5.92	6.10
4	5.90	6.09
8	5.90	6.12
12	5.89	6.13
16	5.87	6.12
20	5.86	6.14
24	5.83	6.16

**Tab. 7:** Stability Analysis in 5% Dextrose

Time (hours)	% of Tramadol HCl	% Degradation at Relative Retention Time							% Total Impurities

		0.27	0.32	0.48	Imp-A	1.14	Imp-C	SA	
0	99.75	0.04	0.04	0.04	0.07	0.06	BDL*	BDL	0.25
2	99.75	0.04	0.04	0.04	0.07	0.07	BDL	BDL	0.25
4	99.76	0.03	0.04	0.04	0.07	0.06	BDL	BDL	0.24
8	99.79	BDL	0.04	0.04	0.07	0.06	BDL	BDL	0.21
12	99.79	BDL	0.04	0.04	0.07	0.06	BDL	BDL	0.21
16	99.79	BDL	0.04	0.05	0.07	0.06	BDL	BDL	0.21
20	99.68	BDL	0.04	0.04	0.07	0.05	BDL	BDL	0.32
24	99.82	BDL	0.02	0.04	0.07	0.05	BDL	BDL	0.18

* BDL: Below Detection Limit (0.025%).

**Tab. 8:** Stability Analysis in 0.9% Sodium Chloride

Time (hours)	% of Tramadol HCl	% Degradation at Relative Retention Time							% Total Impurities

		0.32	0.48	0.65	Imp-A	1.14	Imp-C	SA	
0	99.77	0.04	0.04	0.03	0.07	0.05	BDL*	BDL	0.23
2	99.79	0.04	0.04	0.00	0.08	0.06	BDL	BDL	0.21
4	99.80	0.03	0.04	0.00	0.07	0.06	BDL	BDL	0.20
8	99.80	0.04	0.04	0.00	0.07	0.05	BDL	BDL	0.20
12	99.80	0.03	0.04	0.00	0.07	0.06	BDL	BDL	0.20
16	99.79	0.04	0.04	0.00	0.08	0.05	BDL	BDL	0.21
20	99.79	0.03	0.04	0.00	0.07	0.05	BDL	BDL	0.21
24	99.79	0.04	0.02	0.00	0.07	0.05	BDL	BDL	0.21

* BDL: Below Detection Limit (0.025%).
